# Editorial: Interdisciplinary approaches to enhancing child and adolescent mental health in schools

**DOI:** 10.3389/frcha.2026.1856742

**Published:** 2026-05-12

**Authors:** Oliver Ramos-Álvarez, Víctor Arufe-Giráldez, Alba Ibáñez-García

**Affiliations:** 1 Department of Education, University of Cantabria, Santander, Spain; 2 Research Group on Technology Applied to Research in Employment, Equality and Health (Talionis), University of A Coruña, A Coruña, Spain; 3 Health Economics and Health Services Management Research Group - Marqués de Valdecilla Research Institute (IDIVAL), Santander, Spain; 4 GESTAS Research Group, Exercise Physiology Research, Politécnico Colombiano Jaime Isaza Cadavid, Carrera, El Poblado, Medellín, Colombia

**Keywords:** child and adolescent mental health, implementation science, interdisciplinary approach, physical activity and wellbeing, school environment

## 1 Introduction

Mental health in childhood and adolescence has become established as a global priority in educational and public health systems. This is due to the alarming increase in emotional, behavioural and social problems that appear at increasingly early ages. This phenomenon not only has immediate implications for students' wellbeing, but also conditions life trajectories in the medium and long term. In this sense, mental health must be understood as a multidimensional construct that integrates emotional, psychological and social components that allow children and adolescents to cope with everyday demands, establish meaningful relationships and actively participate in their environment.

The works included in this special issue, “*Interdisciplinary Approaches to Enhancing Child and Adolescent Mental Health in Schools*”, provide a broad and complementary view of the determinants and mechanisms involved in child and adolescent mental health. All of them highlight the need to adopt interdisciplinary approaches that integrate individual, family, school and sociocultural factors, as well as to move towards intervention models oriented towards implementation in real contexts.

## 2 Individual and family factors in child and adolescent mental health

One of the fundamental axes that emerges from the included works is the role of the family environment in the development of psychological wellbeing. Parental involvement, especially in educational activities and early interaction, is often associated with a significant improvement in the child's emotional and behavioural adjustment. It is worth noting that the participation of parents in activities such as shared reading or structured play contributes to the reduction of behavioural problems and the development of socioemotional competencies.

However, these effects are not unidirectional or linear, since evidence has shown that excessive levels of parental involvement can generate the opposite effect, such as increased academic pressure or reduced child autonomy, which can negatively affect psychological wellbeing. This finding underlines the importance of promoting balanced parenting models, based on support, sensitivity and the fostering of autonomy.

In addition, other works in the monograph address the influence of individual factors, such as emotional regulation, motivation or subjective wellbeing, on students' mental health. These studies present a common element, which is the development of socioemotional competencies as a key element for the prevention of psychological problems and the promotion of wellbeing.

## 3 The school environment as a key context for development

The role of the school environment emerges as one of the most consistent factors in explaining mental health in adolescence. The perception of a positive school climate, characterised by supportive relationships among peers and with teachers, as well as by a safe and inclusive environment, is associated with lower levels of psychological symptomatology and greater overall wellbeing.

The results of the included studies show that specific dimensions of the school climate, such as connection with the school, teacher support and perceived safety, act as protective factors against mental health problems. In particular, school connectedness and adult support are identified as significant predictors of lower levels of psychological distress, while environments characterised by low cohesion or limited support may increase the risk of emotional difficulties.

Likewise, physical activity emerges as a relevant element in the promotion of psychological wellbeing. Evidence indicates that a higher frequency of physical activity is associated with better mental health indicators, including lower levels of anxiety, depression and stress. This finding reinforces the need to integrate physical activity into educational policies and school practices as a key strategy for health promotion.

Other works in the special issue deepen the relationship between the school environment and variables such as engagement, motivation or perceived social support, showing that educational contexts that promote participation, inclusion and a sense of belonging contribute significantly to students' wellbeing.

## 4 Multidisciplinary teams and organisation of school mental health services

Beyond the classroom context, the organisation of mental health services in schools constitutes a critical element for student care. Multidisciplinary school mental health teams, composed of professionals from different disciplines, play a fundamental role in the detection, intervention and follow-up of cases.

However, evidence shows that these teams face significant organisational challenges. Among them are the lack of resources, ambiguity in professional roles, inefficient communication and the difficulty in coordinating interventions. These limitations can compromise the quality of services and reduce the effectiveness of interventions.

The work included in this special issue by Albright et al. is a clear example. This work provides a relevant contribution by analysing the adaptation of the TeamSTEPPS model to the school context, highlighting the importance of teamwork training, clarity in roles and organisational leadership. Likewise, it identifies key barriers to implementation, such as the lack of institutional priority or misalignment with school objectives, as well as facilitators such as leadership support and teacher motivation.

Other studies included in the monograph reinforce these findings, indicating that the quality of coordination among professionals and the existence of clear organisational structures are determinants for the success of school mental health interventions.

## 5 Sociocultural perspectives and equity in mental health

A cross-cutting aspect in the included works is the influence of the sociocultural context on child and adolescent mental health. In certain cultural contexts, traditional values can exert both protective and risk effects on psychological wellbeing.

In this sense, some studies in the monograph show that values such as discipline, effort or family cohesion can favour the development of resilience, while norms related to emotional suppression or social pressure may hinder the expression of distress and access to support. This conflict highlights the need to develop culturally sensitive interventions that integrate traditional values with contemporary rights-based approaches.

Likewise, several works highlight the importance of considering equity in access to mental health services, especially in contexts of social vulnerability. Socioeconomic, cultural and territorial inequalities can generate significant barriers to care, which requires the development of specific strategies aimed at reducing these gaps.

## 6 Implementation science: from knowledge to practice

Another relevant contribution of this special issue is the incorporation of implementation science as a framework to understand and improve the transfer of knowledge into practice. This approach recognises that the effectiveness of interventions does not depend only on their design, but also on their capacity to be implemented effectively in real contexts.

The implementation model developed in the work by Albright et al. illustrates how contextual determinants, implementation strategies and outcomes are interrelated, and how the quality of implementation conditions the effects on students. This approach makes it possible to identify barriers and facilitators, as well as to design strategies adapted to the characteristics of the school context.

Other works in the monograph coincide in pointing out that lack of time, teacher overload or resistance to change constitute frequent barriers, while training, institutional support and adaptation to the context act as key facilitators.

## 7 Conclusion

Overall, the works included in this special issue provide an integrative and up-to-date view of interdisciplinary approaches to improving mental health in school contexts. The evidence presented reinforces the idea that students' wellbeing is determined by the interaction of multiple factors, ranging from the family and school environment to organisational systems and sociocultural contexts.

The school is thus consolidated as a privileged space for the promotion of mental health, provided that appropriate organisational structures, trained professionals and coherent and sustainable intervention strategies are in place. In this sense, moving towards interdisciplinary, collaborative and implementation-oriented models constitutes an unavoidable challenge for educational systems.

Finally, this monograph highlights the need to continue developing research that not only generates knowledge, but also facilitates its application in practice, contributing to the construction of healthier, more inclusive and equitable educational environments.

